# Effects of Elastodontic Appliance on the Pharyngeal Airway Space in Class II Malocclusion

**DOI:** 10.3390/jcm12134280

**Published:** 2023-06-26

**Authors:** Assunta Patano, Angelo Michele Inchingolo, Filippo Cardarelli, Alessio Danilo Inchingolo, Fabio Viapiano, Massimo Giotta, Nicola Bartolomeo, Daniela Di Venere, Giuseppina Malcangi, Elio Minetti, Andrea Palermo, Francesco Inchingolo, Gianna Dipalma

**Affiliations:** 1 Department of Interdisciplinary Medicine, University of Bari “Aldo Moro”, 70124 Bari, Italy; assuntapatano@gmail.com (A.P.); drfilippocardarelli@libero.it (F.C.); ad.inchingolo@libero.it (A.D.I.); viapianofabio96@gmail.com (F.V.); massimo.giotta@uniba.it (M.G.); nicola.bartolomeo@uniba.it (N.B.); daniela.divenere@uniba.it (D.D.V.); giuseppinamalcangi@libero.it (G.M.); giannadipalma@tiscali.it (G.D.); 2 Department of Biomedical, Surgical, Dental Science, University of Milan, 20161 Milan, Italy; elio.minetti@gmail.com; 3 College of Medicine and Dentistry, Birmingham B4 6BN, UK; andrea.palermo2004@libero.it

**Keywords:** functional treatment, elastodontic therapy, mandibular retrognathia, Class II malocclusion, airway space, oropharyngeal dimensions, hyoid bone position, breathing disorders, interceptive therapy

## Abstract

Background: The present study analyzed the changes obtained on the upper airway and hyoid bone dimensions in a group of patients with skeletal Class II malocclusion treated with functional elastodontic devices compared to an untreated control group. Methods: A group of 33 patients (19 females and 14 males) with Class II malocclusion treated with AMCOP^®^ SC elastodontic device was compared with a control group of 35 subjects (17 females and 18 males) with untreated Class II malocclusion. Lateral cephalograms were available at the start (T0) and end of treatment/period of observation (T1). Cephalometric analysis was performed and linear measurements to evaluate airway space and hyoid bone position were also obtained. A multivariate analysis of variance for repeated measures (MANOVA) was performed to determine the effects of interactions for the groups for time. Results: Statistically significant differences were found in the study group from T0 and T1 with an improvement of superior upper airway (SPAS *p* < 0.0001), while in the control group it did not change in a statistically significant way from T0 to T1. The MANOVA test showed statistically significant differences between the two groups for the changes of SPAS (*p* = 0.003), IAS (*p* = 0.049), and H-C3 vertical (*p* = 0.038) values. Conclusions: Functional elastodontic therapy produced significant favorable airway changes in skeletal class II subjects.

## 1. Introduction

The configuration and size of the upper airway are determined by anatomical structures such as the soft tissues, muscles, and craniofacial skeleton, which surround the pharynx. Therefore, structural anomalies of the soft tissues and/or craniofacial skeleton can change the pharyngeal airway space [1,2]. The intimate relationship between the stomatognathic apparatus and the first part of the airway allows an understanding of the close anatomical–functional relationship between the two structures. Several studies have analyzed the correlation between airway size and craniofacial features [3,4,5,6,7].

Class II malocclusion is characterized by an alteration in the sagittal plane of the normal relationship of the maxillary bone bases, caused in most cases by hypomandibulia, mandibular retrognathia, or the association of the two conditions [8]. Patients with retrognathic mandibles are more likely to experience a variety of issues, such as an imbalanced facial profile, a retruded chin, lip incompetence, lip trap, related breathing issues [9].

Patients with Class II malocclusion have smaller airway volumes than patients with Class I and III malocclusion [10,11,12,13,14].

According to Balter et al., Class II malocclusion is characterized by a backward positioning of the tongue and a narrowing of the pharyngeal airway space which leads to an altered breathing pattern [15]. When the tongue is positioned backward in patients with retrognathic mandibles, the soft palate is forced backward and the upper airway’s size is reduced [16]. Cozza et al. in 2004 observed that children with respiratory symptoms presented a Class II skeletal pattern with mandibular retrognathia, a deep bite, and the hyoid bone was positioned superiorly [17]. Also, according to Pavoni et al., mandibular retrognathia is associated with a reduced retrolingual space, a reduced diameter of the air-pharyngeal space, and therefore the occurrence of oral respiration [18]. The size of the pharyngeal airway appears to be correlated with the position of the hyoid bone. Because it lacks any bony articulations but is nevertheless suspended by attachments of muscles, ligaments, and fascia of the throat, mandible, and cranium, the hyoid bone is unusual in terms of its anatomic relationship [19]. Another odd aspect of the hyoid is how it moves during various oral processes such as deglutition and respiration. It also has a strong relationship with the tongue due to the genioglossus and geniohyoid’s muscular attachments to it [20].

Patients with Class II skeletal malocclusion are characterized not only by a narrower airway size but also by a more superior and posterior hyoid bone position [21].

In recent years, the effect of orthodontic therapy on the three dimensions of the airway in Class II patients has become a topic of great interest to orthodontists [22,23,24].

Orthopedic therapy using mandibular growth potential is one of the main treatment options in cases of Class II mandibular retrusion. The application of functional devices in the treatment of Class II aims to resolve mandibular retrusion by advancing the mandible and stimulating its growth [25,26,27,28,29].

Functional appliances are a group of active or passive devices that act at the level of the orofacial musculature and transmit forces to the teeth and bone bases, inducing orthodontic and orthopedic changes [30].

The forces produced by these devices, being intermittent, are able to reshape the bone better than continuous forces. All functional appliances used to correct Class II malocclusions have as a common feature the induction of a forward displacement of the mandible. Depending on the devices, the displacement is induced by mucosa-initiated stimuli (with vestibular or lingual shields) or by stimuli originating from obligatory occlusal relationships [31]. Some appliances are attached to dental elements while acting primarily on muscles; others, however, are free in the oral cavity stimulating muscle function whenever they tend to fall out [31].

The mandibular advancement achieved influences the position of the hyoid bone and improves upper airway morphology [32,33,34].

A new approach to functional orthodontic treatment is represented by elastodontic therapy, which uses light and elastic forces, through soft and minimally invasive devices, to correct malocclusions in developmental age by restoring the proper function and harmonious growth of the bone bases [35,36,37].

Elastodontic devices are soft and minimally invasive and require minimal cooperation from young patients. These devices are removable, comfortable, simple in construction and function, and can be used in very young patients without impression taking but by choosing the size based on a simple intraoral measurement or by using classic waxes to detect the size of the arches. They act in a three-dimensional way on all the structures of the stomatognathic apparatus and can correct soft tissue functional problems, promoting restoration of oral, perioral, and lingual muscle function [38,39,40,41].

The results of using elastodontic devices can be superimposed on those of using other functional equipment, such as Fränkel, Bionator, or Twin-Blocks so they can represent a viable alternative [27,42].

The aim of the present study is to analyze the effectiveness of elastodontic therapy using a bioactivator in growing patients with skeletal class II, with particular reference to changes in upper airway dimensions and hyoid bone position, comparing a group of treated patients to an untreated control group.

## 2. Materials and Methods

### 2.1. Sample Selection

The research was approved by the Ethical Committee at the Policlinico of Bari and informed consent was signed by each patient’s parents. A study sample of 33 subjects (14 males and 19 females, mean age 8.9 ± 1.6 years) with Class II malocclusion treated with AMCOP bioactivators was collected from patients referred to the Department of Orthodontics at the Policlinico of Bari and to an orthodontic practice. The treated group was compared with an untreated control group of 35 subjects (18 males and 17 females, mean age 8.9 ± 0.4 years) selected from the archives of the American Association of Orthodontists Foundation Craniofacial Growth Legacy Collection (http://www.aaoflegacycollection.org, Bolton Brush Growth Study, Burlington Growth Study, Iowa Growth Study, Mathews Growth Study, Michigan Growth Study, and Oregon Growth Study, accessed on 1 June 2022). The control group had the same inclusion and exclusion criteria as the treated group.

Inclusion criteria were the following:

- Second skeletal class (ANB > 4°);
- Improvement in facial profile when the lower jaw was placed in an advanced position
- Normo-divergent growth pattern;
- Patients who had had pre- and post-treatment latero-lateral X-rays.

Exclusion criteria were as follows:

- Previous orthodontic treatment;
- Naso-pharyngeal obstructions;
- History of adenoidectomy or tonsillectomy;
- Craniofacial syndromes and abnormalities.

All patients in both treated and untreated groups were at a prepubertal or pubertal stage in skeletal growth at initial observation.

Informed consent was obtained for each patient. For the orthodontic treatment plane the following documentation was collected: extraoral and intraoral photos, orthodontic study models, and panoramic radiographs and latero-lateral teleradiographs at the beginning of treatment/period of observation (T0) and at the end of treatment/period of observation (T1).

### 2.2. Treatment Protocol

The treatment plan involved the use of Multifunctional Cranio-Occlusal-Postural Harmonizer (Armonizzatori Multifunzionali Cranio-Occluso-Posturali, AMCOP) bioactivator, a thermo-activatable elastic device with functional, orthopedic, joint, neuromuscular, occlusal, and postural action.

The AMCOP bioactivators consist of two flanges, vestibular and lingual, which demarcate a free central area that is the balance zone between tongue force and cheek and lip force, in which the teeth go to position themselves without any constraint. The high vestibular flanges perform the dual function of lip-bumper, pushing away the perioral muscles, and proprioceptive stimulation of the bone matrix. They simultaneously involve both dental arches by performing orthopedic action on the maxillary bones in transverse, sagittal, vertical, and torsional directions. The presence of a lingual ramp and tongue button also direct the tongue toward the palate, allowing restoration of proper lingual posture and function. Median incisions were made so as not to interfere with the labial and lingual frenula and the retroincisal papilla. Finally, two concavities at the canine drafts allow for the avoidance of interference with them [35,43].

The devices consist of a polymer/elastomer blend. Two different consistencies of Shore gradation, 51 and 60, are available depending on operational needs. The material is very elastic, thermoplastic, and heat-activatable, and can be adapted to different types of arches. There is also the option of expanding the device by soaking it in hot water at about 70 °C for 30 s and then in cold water to stabilize it in its new shape [38,44].

AMCOP bioactivators exist in different sizes, different colors based on skeletal classes and arch forms, and are designed for deciduous, mixed, and permanent dentition arches, and therefore are suitable for all ages.

**Figure 1:**
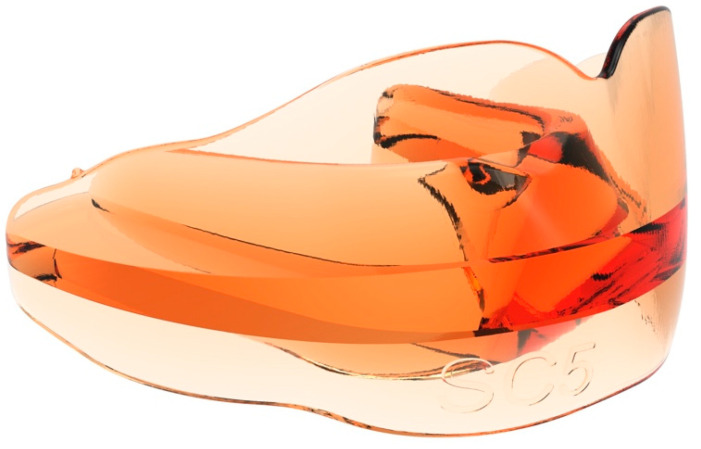
AMCOP SC device.

In this study, patients used AMCOP SC (Figure 1) that is suitable for the treatment of mandibular retrognathia in deciduous, mixed, and permanent dentition. This elastodontic device, unlike most traditional functional braces, does not require an advance construction bite on which to make the device so that occlusal relationships are reproduced in a new position. It is equipped with a mandibular anterior sliding plane that favors mandibular advancement and head-to-head incisor placement, resulting in lengthening of the mandibular bone base and controlling maxilla growth.

Each device is made in different sizes. The measurement of the devices was determined by measuring the transverse distance between the vestibular cusps of the upper first molars [38,44].

**Figure 2:**
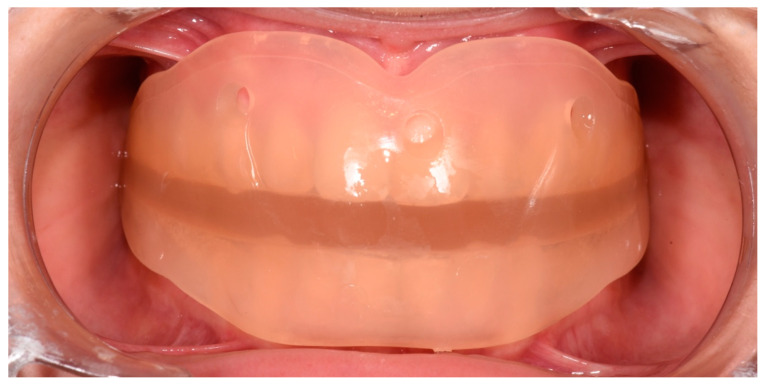
AMCOP SC device worn.

Patients wore the device for 1 h during the day and throughout the night for 6–8 months, and then only at night (Figure 2).

### 2.3. Cephalometric Analysis

For each patient, cephalometric analyses were performed at the beginning of treatment/period of observation (T0) and at the end of treatment (T1). All cephalometric analyses were performed using DeltaDent^®^ 2.5.2. software.

The values obtained from each patient’s measurement were then collected in an excel sheet and subjected to statistical analysis.

**Figure 3:**
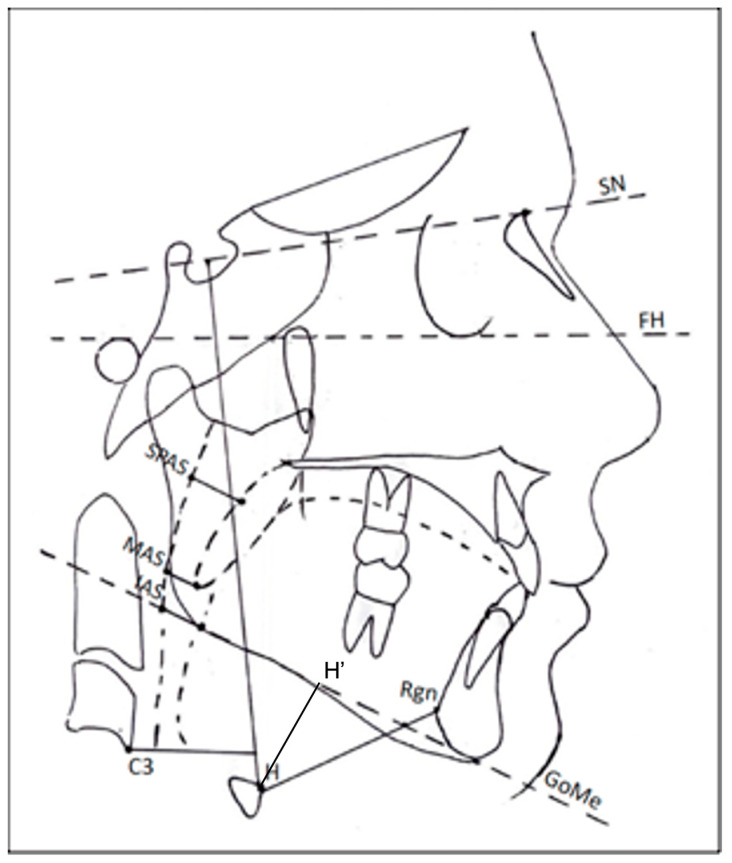
Cephalometric analysis: Airway dimensions [SPAS (mm); MAS (mm); IAS (mm)]; Hyoid bone [H–C3 horizontal (mm); horizontal distance between H and C3; H–C3 vertical (mm); vertical distance between H and C3; H–RGn (mm); distance between H and Rgn; H-H′ (mm): distance between H and the mandibular plane; H–SN (mm); vertical distance from line SN].

The cephalometric parameters considered were:

- Dento-skeletal parameters:

SNA: angle between Sella-Nasion and Nasion-point A segments;

SNB: angle between Sella-Nasion and Nasion-point B segments;

Ans-Pns^Go-Gn: intermaxillary angle, between bispinal plane (Ans-Pns) and mandibular plane (Go-Gn);

SN^Go-Gn: mandibular angle, between Sella–Nasion plane (S-N) and mandibular plane;

FMA: angle between Frankfurt plane and mandibular plane;

Co-Me (mm): distance between the Condilion point and the Menton point;

OVJ (mm): overjet, distance on the sagittal plane between the upper and lower incisors;

OVB (mm): overbite, distance on the vertical plane between the upper and lower incisors;

- Airway size (Figure 3):

SPAS (mm): upper posterior airway space, which is the distance between the soft palate and the posterior wall of the nasopharynx along a line parallel to the Gonion–Menton plane (Go–Me);

MAS (mm): middle airway space, which is the distance between the lower tip of the soft palate and the posterior wall of the oropharynx along a line parallel to Go–Me plane;

IAS (mm): lower airway space (distance between the tongue base along Go–Me plane and the posterior wall of the pharynx);

- Hyoid bone position (Figure 3):

H-C3 horizontal (mm): horizontal distance from H (hyoid, anterior most superior point of the body of the hyoid bone) to C3 (point at anteroinferior position of the third cervical vertebra);

H-C3 vertical (mm): vertical distance from H to C3;

H-Rgn (mm): distance from H to Rgn (retrognathion, lowest posterior point on mandibular symphysis;

H-H′ (mm): vertical distance between H and H′ (projection of H on the Go–Me plane);

H-SN (mm): vertical distance of H to Sella–Nasion plane.

### 2.4. Stastistical Analysis

Continuous variables were expressed as mean and standard deviation (SD) for normally distributed parameters or the median and interquartile range (IQR) in the case of skewed data distribution. Shapiro–Wilk’s statistics were used to test normality. A comparison between Cephalometric measures at baseline (T0) and T1 in each treatment group was analyzed using a Student’s paired *t*-test or nonparametric Wilcoxon signed rank test, while comparison between treatments at each time was performed using Student’s *t*-test or nonparametric Mann–Whitney test. The Chi-square test or Fisher’s exact test, as necessary, were used for the comparison of categorical parameters between treatments.

The independent effect of the treatment on the parameters during the intervention period was tested by a Multivariate Analysis of Variance (MANOVA) for repeated measures; sex, age at baseline, and time between T0 and T1 were used as adjustment variables.

All tests of statistical significance were two-tailed, and *p*-values less than 0.05 were considered statistically significant. Statistical analysis was performed using the SAS/STAT^®^ Statistics, Version 9.4 (SAS Institute Inc., Cary, NC, USA).

## 3. Results

The duration of orthodontic treatment/period of observation was in median 3 years (IQR 2–4).

The main characteristics of the patients at T0 and T1 by the group under analysis are illustrated in Table 1. The study group showed statistically significant differences from T0 and T1 for the following parameters: SNA, SNB, ANB, Co-Me, H-C3 horizontal, H-H′, H-Rgn, H-SN, OVJ, and SPAS. In the control group there was a significant increase in intermaxillary angle, Co-Me, FMA, H-C3 horizontal, H-H′, H-Rgn, and H-SN while ANB reduction and SPAS improvement were not statistically significant.

**Figure 4:**
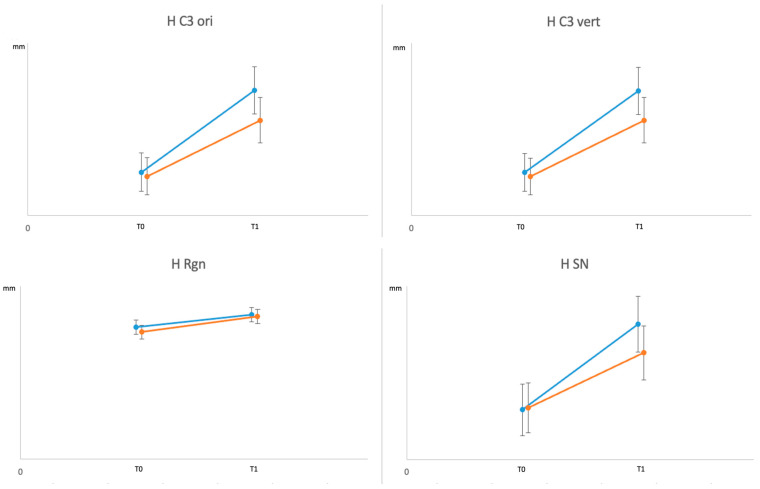

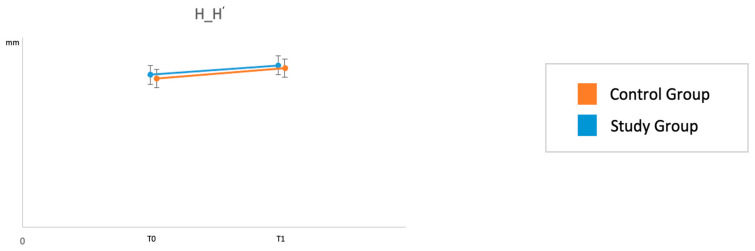
Independent effect of the treatment on the airway parameters during intervention period. In the graph are shown the Lest Square Means and their standard deviation at baseline (T0) and T1.

**Figure 5:**
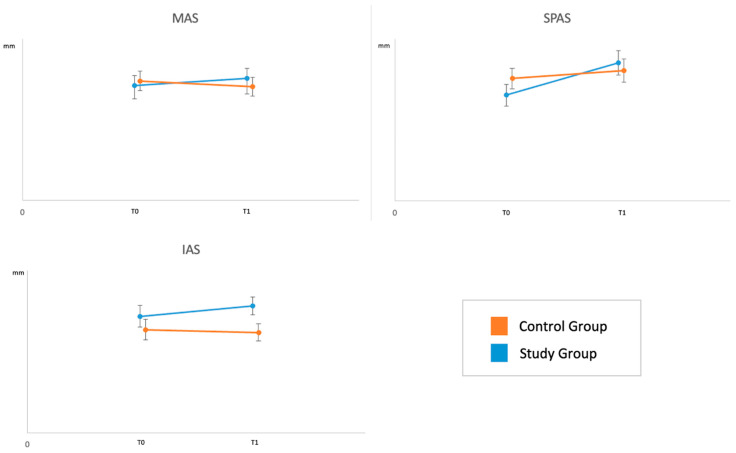
Independent effect of the treatment on the hyoid bone parameters during intervention period. In the graph are shown the Lest Square Means and their standard deviation at baseline (T0) and T1.

The changes of each parameter from T0 to T1 are represented in the graphs in Figure 4 and Figure 5.

Furthermore, evaluating the changes in IAS in the two groups, an increase in the value is observed in the study group with a very low significance although not statistically significant (*p* = 0.0557), while in the control group there was a decrease in this value, even if not statistically significant.

Results from MANOVA statistical analysis are illustrated in Table 2. Statistically significant values were obtained about the effect of the appliance on the variables SNA, ANB, OVJ, OVB, SPAS, IAS, and H-C3 vertical showing a *p*-value below 0.05.

Age and sex factors did not show to have a statistically significant influence on any of the considered parameters. However, the time interval between T0 and T1 resulted in statistically significant changes for H-Rgn and SNB.

Regarding patients’ compliance, the device appeared to be well tolerated. Excessive salivation and pain while waking up with the device all night were common initial issues. Within a few days of starting treatment, these side effects were observed to gradually fade, and, after this time, all children and their parents reported excellent compliance with the MM. The teeth did not exhibit any abnormalities.

## 4. Discussion

The present study analyzed the changes obtained after functional elastodontic treatment of skeletal class II malocclusion with special regard to the effects on the upper airway and hyoid bone.

Although there is still little work in the literature on elastodontic therapy, several studies have previously reported the efficacy of these devices in skeletal class II [45,46,47].

Such devices have been shown to be effective in improving Class II malocclusions, particularly overjet and overbite values, space deficits, and skeletal and molar relationships [48,49,50]. Therefore, the devices can act on sucking, phonation, chewing, swallowing, and breathing by achieving proper naso-laryngo-pharyngeal function [51,52].

The results obtained showed significant changes following treatment, suggesting the effectiveness of the elastodontic appliance in correcting Class II. At the end of treatment, restoration of normal sagittal plane relationships of the maxillary bone bases was obtained, as indicated by the reduction in the ANB. Although the device results in an increase in SNB angle, this value is not statistically significant from the MANOVA analysis when compared to the control group in which there is an increase related to mandibular growth. This result may be related to the sparseness of the sample.

Analyzing the airway data, a significant increase has been found in the sagittal plane of SPAS, and IAS, suggesting an improvement of the airway sagittal space after functional elastodontic treatment. This increase can be related to the fact that the elastodontic device promotes the forward positioning of the mandible, and muscular re-education and tongue advancement [53].

According with our results, Ozbek et al., as early as 1998, found a significant increase in pharyngeal airway in patients treated with a Harvold-type activator compared with an untreated control group [54].

From the 2011 study by Schutz et al.; performed by examining a sample of patients undergoing treatment with maxillary expansion; and Herbst, there emerged as a result of mandibular advancement a shift to a more anterior position of the hyoid bone with consequent forward traction of the tongue. This promoted, therefore, an increase in pharyngeal space with less resistance to the passage of air [55].

A significant increase in airway was, moreover, obtained in another study conducted in a group of subjects with Class II malocclusion from mandibular retrusion treated with Twin-Block [56].

Pavoni et al., on the other hand, compared a group of patients treated with Bionator or Activator to a control group in 2017, concluding that treatment with functional devices produced significant favorable changes during active treatment in sagittal oro and nasopharyngeal airway dimensions in Class II dento-skeletal subjects, and these changes were maintained over the long term [57].

In a retrospective study by Ciavarella et al., the authors evaluated the effects on upper airway and tongue position of a new functional device called SOCIA in hyperdivergent Class II malocclusion patients with mandibular retrusion. This evaluation showed an advancement of tongue position resulting in increased upper airway space. In particular, the most important changes were observed in the SPAS [58].

The same conclusion is reported in a recent study by Inchingolo et al.; regarding the efficacy of elastodontic treatment in hyperdivergent subjects with Class II malocclusion; in which, when also analyzing changes at the airway level, a statistically significant increase in the upper space at the SPAS level was found [59].

Since a narrowed air-pharyngeal space may predispose to oral breathing and obstructive sleep apnea [57], a large number of studies arise from the possibility of preventing or resolving respiratory issues through the use of orthopedic-functional intraoral devices [60].

Maspero et al. in 2015 evaluated by cone-beam CT scan airway changes in 40 patients with skeletal class II obstructive sleep apnea following Andresen activator treatment. In the study group, compared with the control group, significant improvement in soft palate tilt and oropharynx and hypopharynx dimensions was observed following correction of mandibular position in patients treated with the Andresen [61].

Pavoni et al. also conducted a study on pediatric patients with skeletal class II and obstructive sleep apnea syndrome treated with modified Monoblock. This device resulted in significant short-term changes in sagittal airway dimensions and hyoid position compared with untreated controls. In addition, after orthodontic treatment, a significant reduction in daytime symptoms resulted in treated patients [57].

The present study also evaluated the changes obtained in the position of the hyoid bone following the functional elastodontic treatment. Although most values did not change significantly differently in the two groups, in the study group, compared to the control group, there was a more significant increase in the vertical distance from the third cervical vertebra.

The results reported in the literature on changes in the position of the hyoid bone after treatment with Class II functional appliances are controversial.

Odzemir et al. investigated the effects of treatment with a fixed functional appliance on airway dimensions, dentoalveolar relationship, and tongue and hyoid positions and reported that there was no statistically significant change in the hyoid position or the oropharyngeal space [62].

Schutz found that hyoid bone shifts forward but its position remains unchanged vertically [55]. Significant forward displacement of the hyoid bone unaccompanied by vertical displacement has also been described by Babvek et al. [63]. In contrast, another paper that studied the position of the hyoid bone after Twin-Block treatment reported a smaller displacement but the angular position remained unchanged [64].

Ulusoy et al. also reported a change in the position of the hyoid bone downward and forward in the Activator-treated study group compared with an untreated control group [65].

In contrast, Savoldi et al. found no statistically significant change in the position of the hyoid bone, as well as in the size of the airway, in patients treated with Herbst compared with a group treated with high traction [66].

In our study, we compared patients with a control group extrapolated from a historical archive; although this represents a limitation [67,68], for ethical reasons it was not possible to analyze a group of contemporary patients who had not been treated.

Another consideration to highlight is that the use of lateral cephalograms for evaluation purposes, which provide a bi-dimensional picture of a complicated three-dimensional airway, has certain limitations in the current investigation. Infact, a three-dimensional airway assessment would have provided more reliable data by allowing volumetric measurements than the latero-lateral teleradiography we used [69,70]. However, even though lateral cephalograms are not the best instrument for airway analysis, their usage is nonetheless common and airway dimensions on lateral cephalograms were also shown to be highly reproducible [71]. Furthermore, although 3D imaging would be a suitable tool for assessing the oropharyngeal space, it is not widely used and has a risk of rather high radiation exposure. As a result, the traditional lateral cephalogram is still a useful and trustworthy diagnostic tool in many airway studies.

Finally, since we were limited to a purely anatomical assessment, a functional evaluation of the effects of AMCOP^®^ devices on the muscles of the stomatognathic apparatus might be useful. Further research with a long-term follow up is required as it is necessary to assess the long-term stability of the results achieved.

## 5. Conclusions

Skeletal class II malocclusion can be corrected using the AMCOP^®^ SC that promotes mandibular advancement. Furthermore, the present study demonstrated that functional elastodontic device therapy results in significant airway changes in skeletal class II subjects, compared with an untreated control group, and led to an improvement of deglutition, phonation, and respiratory function. In addition, the hyoid bone shifted inferiorly at the end of treatment in the treated group with respect to the control group.

Further studies are needed regarding the effects of elastodontic treatment, which could provide a comfortable and minimally invasive interceptive treatment option that would allow restoration of proper oropharyngeal neurovegetative function, preventing the onset of respiratory problems.

## 6. Patents

 Title: dispositivo ortodontico-elastico-armonizzatore dento cranio facciale, scope: Italian, granted under n. 102015000057082.

 Title: dispositivo ortodontico-elastico-armonizzatore dento cranio facciale, scope: International, granted under n. WO 2017/056010.

## Figures and Tables

**Table 1:** Main characteristics of the patients at baseline and at time T1.

		CASE			CONTROL		Comparison at T0
	T0	T1	*p*-Value	T0	T1	*p*-Value	*p*-Value
SNA	82.5 ± 2.6	81.5 ± 2	0.0053	80.8 ± 2.7	81.2 ± 3.5	0.2515	0.0093
SNB	76.7 ± 2.3	78 ± 2.2	<0.0001	74.9 ± 2.5	75.5 ± 3	0.0617	0.0029
ANB	5.8 ± 1.4	3.6 ± 1.2	<0.0001	5.9 ± 1.5	5.7 ± 2.1	0.5204	0.8423
Co_Me	92.4 ± 6.3	100.9 ± 7	<0.0001	91.2 [88.9–95.8]	96.6 [92.3–100.1]	<0.0001 †	0.7777 *
FMA	23.1 ± 3.4	22.3 ± 3.4	0.0773	23 ± 2.8	22.1 ± 3	0.0221	0.9084
Pns-Ans^Go-Gn	23.3 ± 3.9	22.5 ± 4.6	0.1039	24.4 ± 3.5	23.1 ± 4	0.0036	0.2525
SN^Go-Gn	31.6 ± 3.7	31.4 ± 4.1	0.5227	32.5 ± 3.1	32.1 ± 3.7	0.2062	0.2776
OVB	2 ± 1.9	2.3 ± 1.1	0.5079	1.9 [0.4–3.6]	3.3 [2.4–4.4]	0.0001 †	0.6540 *
OVJ	5.3 [4.37–6.65]	3.2 [2.65–3.55]	<0.0001 †	5.6 [4.4–6.8]	5.8 [4.4–6.4]	0.2132 †	0.6364 *
H_C3_oriz	27.8 [26.0–30.3]	29.9 [27.5–32.6]	0.0118 †	27.6 ± 3.1	29.4 ± 3.5	0.0006	0.5061
H_C3_vert	2.8 ± 6.3	3.6 ± 5.1	0.3272	4.7 ± 4.9	3.5 ± 5.6	0.0797	0.174
H_H’	10.6 ± 5.6	12.5 ± 5.7	0.0027	10.6 ± 4.3	13 ± 4.5	0.0005	0.9854
H_Rgn	30.5 ± 5.1	33.7 ± 5.2	0.0061	29.4 ± 5.1	32.9 ± 5.1	<0.0001	0.3619
H_SN	85 ± 8.9	93.8 ± 9	<0.0001	85.4 ± 7.3	91.1 ± 8.4	<0.0001	0.8076
IAS	11.5 ± 3.4	12.5 ± 2.6	0.0557	10.1 ± 2.7	9.9 ± 2.3	0.4482	0.0776
MAS	10 ± 3	10.6 ± 2.8	0.1989	10.3 ± 2.3	9.9 ± 2.3	0.1709	0.6131
SPAS	9.3 ± 3.1	12.1 ± 3.2	<0.0001	10.6 ± 2.9	11.2 ± 3.3	0.1772	0.0759

Data are shown as mean ± standard deviation or median [IQR]; † *p*-value calculated with Wilcoxon signed-rank test; * *p*-value calculated with Mann–Whitney Test.

**Table 2:** Least Square Means of the parameters from a repeated measure multiple analysis of variance.

	CASE		CONTROL		Effect from T0 to T1 (*p*-Value)			
Parameter	T0	T1	T0	T1	Group	AGE_T0	Time	Sex
SNA	82.4 ± 0.5	81.5 ± 0.5	80.8 ± 0.4	81.3 ± 0.5	0.008	0.879	0.496	0.545
SNB	76.7 ± 0.4	77.9 ± 0.4	74.9 ± 0.4	75.5 ± 0.4	0.102	0.422	0.027	0.456
ANB	5.8 ± 0.3	3.5 ± 0.3	5.9 ± 0.2	5.7 ± 0.3	<0.001	0.319	0.196	0.87
Co_Me	92.5 ± 1	101.1 ± 1.2	92.1 ± 1	98 ± 1.2	0.102	0.165	0.128	0.347
FMA	23 ± 0.6	22.2 ± 0.6	23 ± 0.5	22.1 ± 0.5	0.915	0.987	0.235	0.57
Ans-Pns^Go-Gn	23.3 ± 0.6	22.4 ± 0.7	24.4 ± 0.6	23.1 ± 0.7	0.612	0.239	0.214	0.162
SN^Go-Gn	31.7 ± 0.6	31.3 ± 0.7	32.5 ± 0.6	32 ± 0.7	0.8	0.745	0.397	0.072
OVB	2.1 ± 0.3	2.3 ± 0.2	2 ± 0.3	3.5 ± 0.2	0.01	0.393	0.263	0.449
OVJ	5.5 ± 0.3	3.2 ± 0.3	5.9 ± 0.3	5.9 ± 0.3	<0.001	0.756	0.802	0.994
H_C3_oriz	28.2 ± 0.6	29.9 ± 0.6	27.5 ± 0.5	29.4 ± 0.6	0.899	0.285	0.46	0.695
H_C3_vert	2.9 ± 1	3.8 ± 0.9	4.7 ± 0.9	3.5 ± 0.9	0.038	0.414	0.166	0.343
H_H’	10.6 ± 0.9	12.4 ± 0.9	10.6 ± 0.8	13 ± 0.8	0.569	0.337	0.363	0.313
H_Rgn	30.5 ± 0.8	33.4 ± 0.8	29.3 ± 0.8	32.9 ± 0.8	0.592	0.307	0.046	0.092
H_SN	85.1 ± 1.4	94.1 ± 1.5	85.3 ± 1.3	91.1 ± 1.4	0.051	0.676	0.21	0.169
IAS	11.5 ± 0.5	12.5 ± 0.4	10.1 ± 0.5	9.9 ± 0.4	0.049	0.36	0.616	0.591
MAS	10 ± 0.4	10.6 ± 0.4	10.4 ± 0.4	9.9 ± 0.4	0.066	0.432	0.325	0.895
SPAS	9.1 ± 0.5	12 ± 0.5	10.6 ± 0.4	11.3 ± 0.5	0.003	0.428	0.842	0.82

Data are shown as Mean and Standard Error; LT = long of treatment.

## Data Availability

Not applicable.
